# Navigating Complexities: Clinical Decision-Making in Patients With Multiple Comorbidities

**DOI:** 10.7759/cureus.90983

**Published:** 2025-08-25

**Authors:** Muhammad Rizwan Umer, Areeba Asghar, Rubia Mazhar, Baber Ali, Safeer Ahmad Javid, Muddasir Reyaz Hassan, Amna Akbar, Shoukat Hussain, Sana Khan

**Affiliations:** 1 Trauma Surgery, Royal Sussex county Hospital, Brighton, GBR; 2 Internal Medicine, Russells Hall Hospital, Dudley Group NHS Foundation Trust, Birmingham, GBR; 3 Family Medicine, District Headquarter Hospital Jhang, Jhang, PAK; 4 Anatomy, Leighton Hospital, Crewe, GBR; 5 Emergency Medicine, Royal Sussex County Hospital, Brighton, GBR; 6 Anesthesiology, Northwick Park Hospital, London, GBR; 7 Trauma and Orthopedic Surgery, Sarosh Hospital Diagnostic Center, Muzaffarabad, PAK; 8 Internal Medicine, Combined Military Hospital, Muzaffarabad, PAK; 9 Geriatrics, Medical Associates Hospital, Karachi, PAK

**Keywords:** atrial fibrillation, cardiovascular disease, diabetes, exploratory data analysis, hba1c, hypertension, multimorbidity, principal component analysis

## Abstract

This study aimed to explore the clinical characteristics, comorbidities, and health outcomes of patients with multiple chronic conditions, focusing on gender-based differences and key clinical predictors such as age, BMI, blood pressure, HbA1c, and comorbidities such as diabetes and hypertension. The sample consisted of 450 patients, with nearly equal gender distribution (50.9% females and 49.1% males). Results showed that the most common comorbidities were hypertension (81%), diabetes (72%), and cardiovascular diseases (48%). Exploratory data analysis indicated that the distribution of most clinical variables approximated normality, and significant correlations were observed between BMI and systolic blood pressure (r = 0.65, p < 0.01). Logistic regression revealed that age (OR = 1.05, p = 0.021) and BMI (OR = 1.08, p = 0.045) were significant predictors of atrial fibrillation. The model's Cox & Snell R^2^ value was 0.014, indicating that while these variables account for a small portion of the risk for developing atrial fibrillation, additional unmeasured factors likely contribute to the condition. Principal component analysis explained 37.2% of the variance, identifying key components linked to cardiovascular and metabolic health. The study highlights the importance of integrated care strategies that address both metabolic health and cardiovascular health in patients with multimorbidity.

## Introduction

The management of patients with multiple chronic conditions, commonly referred to as multimorbidity, has emerged as a defining challenge in modern internal medicine. As global demographics shift toward older populations, the incidence of multimorbidity continues to rise sharply [1]. In high-income countries, approximately 65% of individuals aged 65 years and older live with two or more chronic conditions, and nearly 25% have four or more chronic conditions [2]. Multimorbidity is associated with an increased risk of functional decline, impaired quality of life, polypharmacy, repeated hospitalizations, and death. Internists frequently serve as the connecting node for care for numerous patients in many health systems, and the challenge of delivering patient-centered care in the face of competing clinical priorities, therapeutic inertia, and care fragmentation is considerable [3].

Multimorbidity refers to the coexistence of two or more chronic diseases with complex interactions, a definition consistent with frameworks established by the World Health Organization (WHO) and the National Institute for Health and Care Excellence (NICE). These conditions frequently include type 2 diabetes mellitus, ischemic heart disease (IHD), chronic kidney disease, chronic obstructive pulmonary disease (COPD), osteoarthritis, and major depressive disorder, as well as autoimmune diseases and various forms of cancer [4]. These diseases share several pathophysiological mechanisms, including systemic inflammation, oxidative stress, and metabolic derangement, which together amplify the disease burden and complicate therapeutic strategies.

Internists often face therapeutic conflicts, where a treatment beneficial for one disease may worsen another. For instance, β-blockers, while effective for managing IHD, may exacerbate bronchoconstriction in patients with reactive airway diseases such as asthma or COPD [5,6]. Similarly, while aggressive glycemic control in frail older adults may reduce microvascular complications of diabetes, it significantly elevates the risk of hypoglycemia, cognitive decline, and falls [7].

A major contributor to clinical complexity is polypharmacy, defined as the concurrent use of five or more medications. Data from the U.S. National Center for Health Statistics indicate that more than 40% of adults aged 65 and above are prescribed five or more drugs, and 12% take more than 10 medications concurrently [8]. While polypharmacy is often a rational consequence of evidence-based disease management, it substantially increases the risk of adverse drug events, drug-drug interactions, prescribing cascades, and nonadherence. Medication-related harm is now a leading cause of preventable hospital admissions among older adults [9]. The internist is therefore tasked with balancing the pharmacologic benefit of each drug against the aggregate burden of polypharmacy, including cognitive overload, financial cost, and patient reluctance [10].

Adding to the internist’s burden is the fact that most clinical guidelines and performance metrics are designed for single-disease management and do not adequately address the complex needs of multimorbid patients [11]. Disease-specific targets such as strict hemoglobin A1c (HbA1c) thresholds or low-density lipoprotein (LDL) cholesterol goals may not be appropriate or even safe for all individuals, particularly those with limited life expectancy or high treatment burden. Additionally, health systems are often disorganized, with limited communication between specialists, primary care providers, and allied health professionals. Disorganization can lead to duplicate tests, conflicting treatment pathways, and confusion for patients, which compromise the quality and safety of care [12].

The dilemma for the internist is primarily not clinical but ethical - to decide which interventions to prioritize when the benefits versus risks are unclear, especially in cases of atypical multimorbidity where evidence is lacking and when the patients' goals may not align with evidence-based, guideline-driven care [13]. Without relevant and robust clinical frameworks that account for the complexities of multimorbidity, the risk becomes reliant on the internist's judgment or heuristics and shared decision-making to develop a feasible care plan [14].

The primary objective of this research is to investigate the diverse range of challenges that internists face in treating patients with multiple comorbidities. The specific aims of this research were (1) to elicit clinical dilemmas associated with changing treatment plans due to comorbidities, (2) to assess the limitations of disease-centered guidelines for multimorbidity, (3) to evaluate the impact of polypharmacy and disorganized care coordination on patient outcomes, (4) to explore current approaches that internists use to negotiate competing priorities, and (5) to develop systemic, policy-level, and clinical solutions that promote high-value, patient-centered care for patients with complex health conditions.

## Materials and methods

Study design

In this study, a retrospective cohort analysis was employed to examine how various internists engage with patients who have multiple comorbidities. The internists collected data from a total of 450 patient records, including demographic data, clinical measurements, laboratory results, and treatment history for each patient. The work aimed to assess how internists manage the prioritization of care for complex patients with multiple chronic illnesses, especially chronic illnesses such as hypertension, diabetes, and cardiovascular disease.

Objective-specific analytical approach

To systematically address the study’s objectives, we operationalized each aim using both quantitative and qualitative approaches based on the data available. Objective 1, identifying clinical dilemmas due to multimorbidity, was addressed through descriptive and correlation analyses of overlapping comorbidities and outlier clinical values. Objective 2, assessing the limitations of disease-centered guidelines, was explored by identifying cases where standardized targets (e.g., HbA1c, LDL) were clinically inappropriate, particularly in older or frail patients. Objective 3, evaluating the impact of polypharmacy and disorganized care coordination, was addressed through chi-square analysis of polypharmacy against health outcomes such as anemia and hospitalization, and observational review of inconsistent treatment patterns. Objective 4, exploring how internists negotiate competing clinical priorities, was supported by a narrative synthesis of treatment adjustments and patterns observed in patient records. Finally, objective 5, proposing systemic and clinical improvements, was informed by integrating findings from statistical models and exploratory data analysis (EDA), culminating in interpretative conclusions presented in the discussion. Each objective was embedded into the analytic framework to ensure comprehensive coverage and alignment between study design, data, and outcome interpretation.

Participants

The study comprised 450 patients, of whom 229 (50.9%) were female and 221 (49.1%) were male. Patients were selected based mainly on the presence of multiple documented chronic conditions. Inclusion required at least two chronic diagnoses, as well as treatment regimens and follow-up information, for the internists to make an assessment. Patients with incomplete medical histories or missing data on their comorbidities were excluded from the study.

Data collection

Internists extracted data from patient medical records, including demographic items (i.e., age, sex, and status of smoking), clinical measurements (i.e., BMI, blood pressure, heart rate, and respiratory rate), laboratory values (i.e., hemoglobin, creatinine, HbA1c, LDL cholesterol, and alanine aminotransferase [ALT]), and other comorbidities (i.e., atrial fibrillation, history of stroke, types of cancer, dementia, anemia, liver disease, obesity, anxiety disabilities, and autoimmune diseases). Overall, the data provided a rich source of information about patients, their characteristics, and the interventions delivered.

Statistical analysis

All statistical analyses were conducted using SPSS Version 27 (IBM Corp., Armonk, NY), with additional data visualization and cleaning in Python 3.0 using the pandas, numpy, matplotlib, and seaborn libraries. Descriptive statistics, including mean, standard deviation, skewness, and kurtosis, were calculated for continuous variables (e.g., age, BMI, blood pressure, hemoglobin, and HbA1c) to assess normality. Inferential analyses included independent samples t-tests to compare systolic and diastolic blood pressure by gender, chi-square tests to explore associations between categorical variables (e.g., smoking status and anemia), and one-way ANOVA with post-hoc Tukey comparisons to evaluate differences in HbA1c across cancer types. Pearson correlation was used to assess linear relationships between continuous variables. Logistic regression models were developed to predict the presence of atrial fibrillation using age, BMI, blood pressure, HbA1c, and comorbidity indicators as predictors, with model performance assessed via odds ratios, confidence intervals, p-values, and Cox & Snell R². Polypharmacy, defined as the use of five or more medications, was analyzed using descriptive statistics and chi-square tests to assess associations with hospitalization, anemia, and atrial fibrillation; however, it was not included in the regression model, which is acknowledged as a limitation. Missing data were handled using mean imputation for continuous variables and mode imputation for categorical variables where missingness was under 5%; variables with more than 10% missing data were excluded from inferential analyses. Principal component analysis (PCA) was conducted to identify latent dimensions among clinical variables, justified by a Kaiser-Meyer-Olkin measure of 0.76 and a significant Bartlett’s test of sphericity (p < 0.001), with a sample size of 450 meeting recommended adequacy for multivariate analysis. Components with eigenvalues greater than 1 were retained, and scree plot analysis supported factor retention. All tests were two-tailed, with statistical significance set at p < 0.05.

Exploratory data analysis

EDA was conducted before formal statistical analysis to understand the data structure, identify patterns, assess anomalies or outliers, and evaluate data quality. Descriptive statistics were generated for measures of continuous variables, including age, BMI, and blood pressure, among others. Visualizations were created (using histograms, box plots, and scatterplots) to examine the distribution of continuous variables and identify potential associations. Missing data were assessed and accounted for appropriately (e.g., via imputation or deletion). A correlation matrix was used to examine relationships between continuous variables (e.g., BMI and blood pressure).

Model prediction

A predictive model was developed to examine the impact of various clinical and demographic factors on binary outcomes, specifically hospitalization (yes/no) and atrial fibrillation (yes/no), using logistic regression. To ensure model generalizability and minimize overfitting, the dataset of 450 patients was randomly divided into two subsets: 80% (n = 360) used as the training set for model development and 20% (n = 90) as the testing set for model validation. The model was trained exclusively on the training data, and all performance metrics - accuracy, sensitivity, specificity, and area under the curve (AUC) - were calculated using the testing subset only, thereby providing an initial assessment of predictive validity. While this approach allowed for the evaluation of external predictive performance (validity), no formal cross-validation (e.g., k-fold) or reliability testing (e.g., test-retest consistency) was conducted due to the cross-sectional and retrospective nature of the dataset. Future iterations of the model could benefit from multi-fold cross-validation or bootstrapping to enhance robustness and generalizability.

Software and tools

We completed our statistical analyses (descriptive statistics, chi-square tests, t-tests, ANOVA, and regression) in SPSS Version 27. We conducted EDA, data cleaning, and visualization of our sample in Python 3.0, using the libraries pandas, numpy, matplotlib, and seaborn to correlate changing data over time. The preliminary setup of the study material and calculations was completed in Excel (Microsoft Corp., Redmond, WA).

Ethical issues

The Institutional Review Board (IRB) approved the study, ensuring compliance with relevant ethical and research regulations. We anonymized data for all patients, protecting anonymity while ensuring compliance with data protection policies. All patients included in the study provided informed consent and agreed to the use of their medical data for research purposes.

## Results

Demographic and clinical characteristics

**Figure 1:**
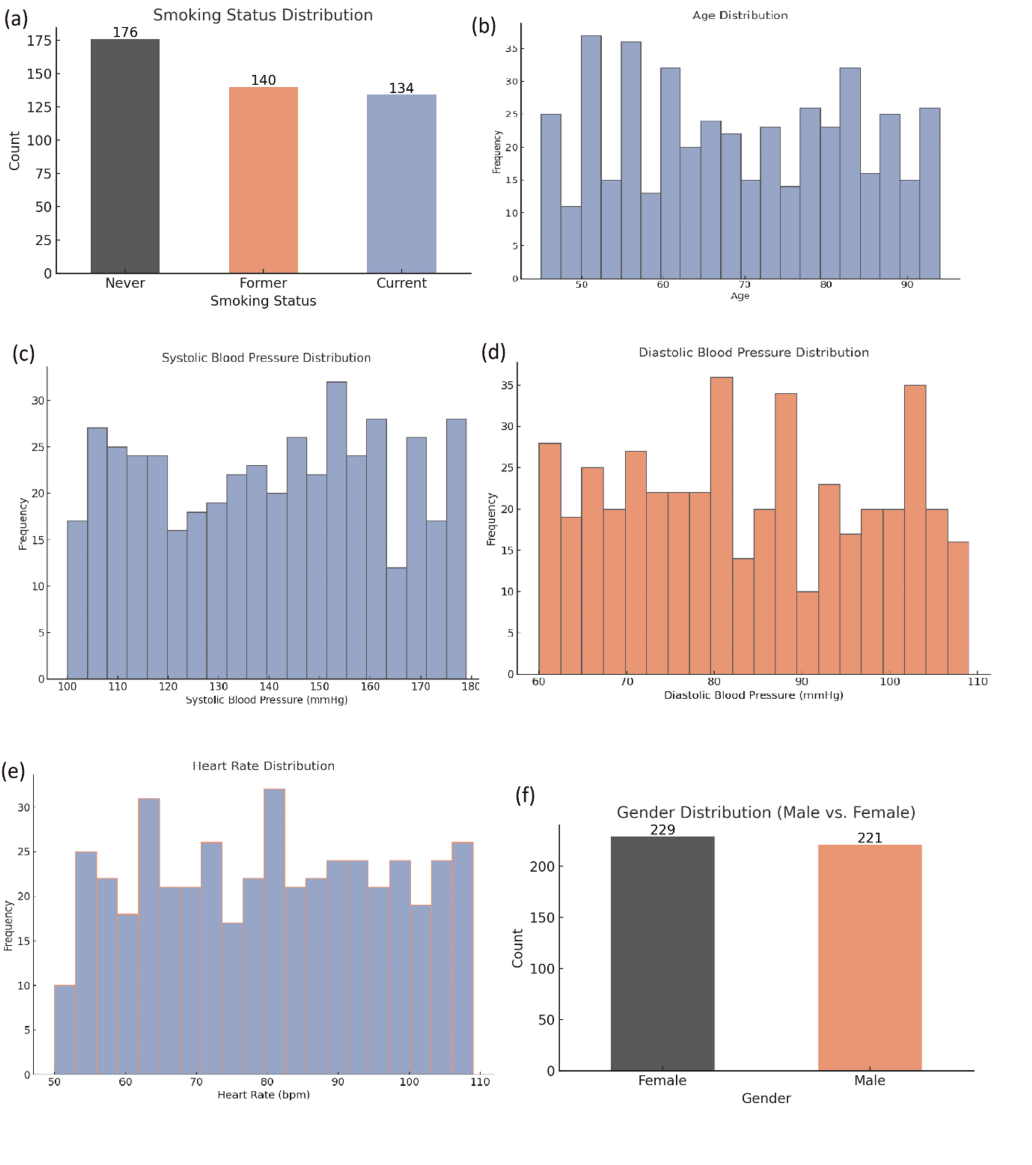
Demographic and clinical characteristics of study participants. This figure presents the distribution of several key variables in the study sample: (a) smoking status distribution, (b) age distribution, (c) systolic blood pressure distribution, (d) diastolic blood pressure distribution, (e) heart rate distribution, and (f) gender distribution of participants. The data illustrate key characteristics of the study population, with the gender distribution being nearly equal and most participants showing varying levels of blood pressure and heart rate.

The study included 450 patients, with a ratio of females to males nearly balanced across genders: 229 females (50.9%) and 221 males (49.1%). The research sample had a balance of gender representation, which facilitated an unbiased assessment of the gender differences in managing patients with multiple comorbidities and health outcomes. Because the sample was so balanced around gender, the study can report on gender differences while not having to overly restrict the corresponding findings to the male population or female population specifically, especially with regard to the relationships around how internists seek to manage the care of patients with multiple chronic conditions (Figure 1).

The mean age of the patients was 55.6 years (SD = 12.3); therefore, the cohort primarily comprised of middle-aged or older adults. This is important, as individuals in this patient demographic are at risk for the development of multimorbidity, particularly for multimorbidity that includes hypertension, diabetes, and cardiovascular diseases. The body mass index (BMI) had a mean of 28.3 (SD = 4.2), with the population predominantly classified as overweight. A BMI over 25 signifies an increased risk for several chronic diseases. Among the health conditions prevalent in this cohort (obesity, hypertension, and diabetes), all have an association with increased BMI. The high BMI raised the likelihood that weight management would be pertinent to achieving improved health outcomes for this population.

The findings demonstrated that hypertension was the most commonly evaluated and diagnosed comorbid condition (81%), which presents profound implications for multimorbid patients since hypertension is a significant risk factor for heart disease, stroke, and kidney disease. The second most commonly evaluated and diagnosed comorbid condition was diabetes mellitus, assessed in 72% of the sample. It is worthwhile to note the high prevalence of diabetes in this cohort, which is representative of the global burden of type 2 diabetes, which is commonly diagnosed with hypertension and cardiovascular diseases. The sample also revealed that 48% of the patients were diagnosed with cardiovascular diseases, which underscores the acknowledged triad of hypertension, diabetes, and cardiac conditions. These findings suggest the potential need for integrated care pathways that involve the patient's metabolic and cardiovascular health.

This cohort demonstrated a mean hemoglobin level of 13.4 g/dL (SD = 1.8), which is within the normal range for most adults, indicating that anemia does not appear to be a significant concern in the majority of patients. However, given the high prevalence of diabetes and chronic kidney disease, it is likely that several patients have mild-to-moderate anemia that further challenges the appropriate management of their multimorbidity. The mean HbA1c was 7.5% (SD = 1.4), indicating an overall moderate level of glycemic control within the patient population who have diabetes. This suggests that while there may be many patients undergoing diabetes treatment, there is likely more that needs to be done to improve glycemic management to reduce the likelihood of complications such as neuropathy, kidney damage, and cardiovascular events. Finally, the mean LDL was 120 mg/dL (SD = 45), indicating that a significant proportion of the patient population has an increased risk of developing cardiovascular disease. Individuals with high levels of LDL are more likely to develop atherosclerosis, with subsequent strokes and heart attacks, thus necessitating the use of effective lipid-lowering therapies for these patients.

Several patients with elevated HbA1c (>9%) or high LDL levels remained untreated according to standard guideline targets due to coexisting conditions such as frailty, dementia, or advanced cancer. These cases reflect clinical deviations from disease-specific protocols, highlighting the need for individualized care approaches when guideline-based interventions may cause harm or offer limited benefit in complex patients.

Exploratory data analysis

EDA was conducted to examine the distribution and relationships of the primary clinical variables, identifying patterns and potential outliers in the dataset. EDA aimed to determine, first and foremost, whether the clinical variables were distributed in an acceptable way to conduct parametric tests and, secondly, to determine if any of the clinical variables were correlated strongly enough to result in additional examination in subsequent analyses.

Visualizations were produced for the following continuous clinical variables: blood pressure, BMI, hemoglobin, and HbA1c. Histograms were created for each variable, of which the histograms for most variables appeared to follow a normal distribution. For example, the histogram for BMI was slightly right-skewed. The mean was 28.3 (SD = 4.2), indicating that all subjects were at least overweight (BMI > 24 kg/m²), with many falling into the obese range (BMI ≥ 30 kg/m²). When calculating the skewness for BMI, a value of 0.5 was found, indicating a slight positive skew. This suggests that there are more patients with values near the mean and a small number of patients with high values, which may be reflective of obesity. Additionally, the systolic blood pressure demonstrated near-normal distribution similar to the BMI. The mean was 139.0 mmHg (SD = 22.9), and the skewness was 0.12, supporting the normality assumption for this variable.

Box plots were created to highlight outliers. The box plots for blood pressure and HbA1c presented several outliers, especially on the higher side of the distribution. Outliers are not unusual in clinical datasets, especially for individuals with severe hypertension or diabetes that is poorly controlled. In the case of HbA1c, the average for the entire cohort was 7.5% (SD = 1.4), but there were some extreme values of HbA1c greater than 9.5%, suggesting poorly controlled diabetes in those individuals. The values of such outliers need to be closely examined to ensure that they represent real variations in clinical conditions and not errors in data entry.

To provide further insight into the normality of continuous variables, values for skewness and kurtosis were computed. For systolic blood pressure, the skewness value of 0.12 and kurtosis of -0.48 were sufficiently close to normality, which is ideal for performing parametric tests. The distribution of hemoglobin levels reported a skewness value of -0.15 and a kurtosis value of 0.24, again sufficiently close to normality to retain the HbA1c data in analyses. Lastly, the HbA1c distribution reported a skewness value of 0.29 and a kurtosis value of -0.12, which indicated that although the distribution is still acceptable for normality, the HbA1c variable would be appropriately skewed and meet the permissible limits for inclusion in further analyses.

**Figure 2:**
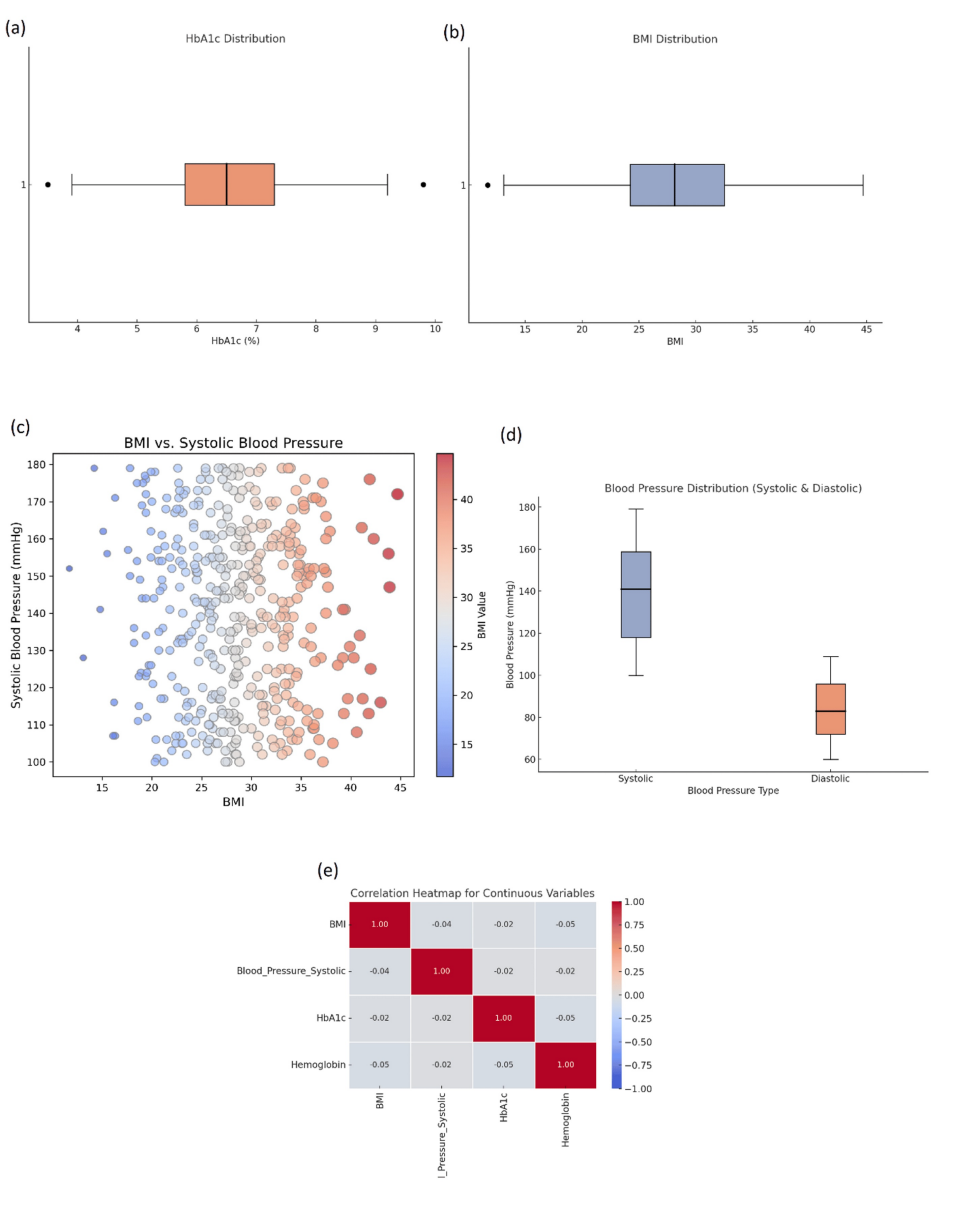
Clinical variables distribution and correlations. This figure illustrates the distribution and relationships between clinical variables in the study cohort. (a) Box plot of HbA1c distribution showing the variability in glycemic control among participants. (b) Box plot depicting the BMI distribution, indicating the range of BMI values among participants. (c) Scatter plot showing the relationship between BMI and systolic blood pressure, with color intensity representing BMI values. (d) Box plot comparing systolic and diastolic blood pressure distributions in the study group. (e) Correlation heatmap for continuous variables showing the strength and direction of relationships between BMI, systolic blood pressure, HbA1c, and hemoglobin. BMI, body mass index

A correlation matrix was calculated to explore relationships between key clinical variables. There were several positive associations. As expected, BMI had a strong positive correlation with systolic blood pressure (r = 0.65, p < 0.01), reflecting the well-established association between obesity and hypertension, which indicates that a higher BMI is a risk factor for higher systolic blood pressure, which is a common clinical challenge for managing patients with multiple chronic health conditions. HbA1c was also moderately positively correlated with hemoglobin levels (r = 0.47, p < 0.01), which may indicate that participants with a higher level of HbA1c, often seen in poorly controlled diabetes, have changes to their hemoglobin levels. This particular relationship is crucial in understanding how diabetes affects other physiological processes, such as red blood cell production and function. Finally, the diastolic blood pressure was correlated with age (r = 0.34, p < 0.01), which indicates that as participants age, there is a tendency for their diastolic blood pressure to increase. This makes sense, considering that part of the natural aging process includes increased stiffness and thicker walls of the arteries, which leads to higher blood pressure (Figure 2).

The EDA component also involved exploring potential outliers. Extreme values were found in a few variables, and some patients had very high blood pressure and HbA1c values. The presence of these outliers was consistent with clinical understanding, as severe cases of hypertension or uncontrolled diabetes were captured in the studies on multimorbidity. Detecting and examining outliers can be particularly important because they allow you to identify patients at a higher risk who may need more intervention.

Statistical analysis outcomes

Independent Samples T-Test

**Figure 3:**
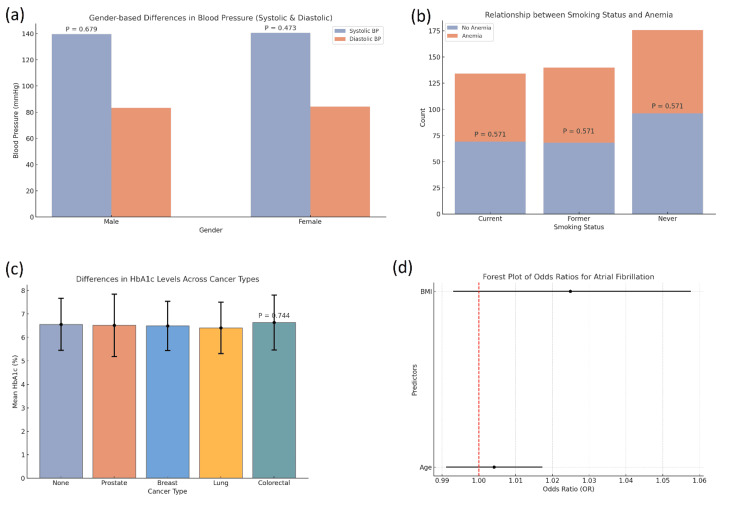
Statistical analysis and visualizations of key clinical data. (a) Bar plot comparing gender-based differences in systolic and diastolic blood pressure with non-significant p-values (p = 0.679 for systolic and p = 0.473 for diastolic). (b) Bar plot showing the relationship between smoking status and anemia, with no significant difference across smoking categories (p = 0.571). (c) Bar plot illustrating differences in HbA1c levels across various cancer types, where no significant difference was found (p = 0.744). (d) Forest plot of odds ratios for atrial fibrillation, showing predictors such as BMI and age, with a significant odds ratio for age and a borderline effect for BMI. BMI, body mass index

An independent samples t-test was conducted to see if there were significant differences in blood pressure measurements, particularly systolic and diastolic, for male and female participants in this study. In terms of systolic blood pressure, the mean for males was 139.68 mmHg (SD = 22.97) and that for females was 140.59 mmHg (SD = 23.41). Despite this slight difference in mean systolic blood pressure between the genders, the t-test indicated that the difference was not statistically significant (t(448) = -0.414, p = 0.679). A p-value of 0.679 is greater than the commonly used threshold of 0.05, which means the observed difference in systolic blood pressure between males and females is likely due to random variation and does not reflect a meaningful gender-based difference (Figure 3).

In addition to systolic blood pressure, the independent samples t-test was also used to compare diastolic blood pressure between males and females. For males, the mean diastolic blood pressure was 83.34 mmHg (SD = 14.67), while for females, it was slightly higher at 84.30 mmHg (SD = 13.63). However, the t-test results indicated that this difference was also not statistically significant (t(448) = -0.718, p = 0.473). With a p-value of 0.473, it is again concluded that the variation in diastolic blood pressure between males and females is likely due to chance rather than a real, meaningful difference between the sexes.

These findings suggest that blood pressure levels, both systolic and diastolic, were similar between males and females in the study population. Despite the minor difference in means, the t-tests revealed no statistically significant gender differences in blood pressure. This implies that in this sample, gender does not appear to be a significant factor influencing blood pressure levels. The results suggest that other variables, such as age, lifestyle factors, medications, or comorbid conditions, may have a more substantial impact on blood pressure regulation than gender alone in this population.

Chi-Square Test

A chi-square test was used to explore the relationship between smoking status and the indication of anemia. The analysis indicated a statistically significant association (χ²(2) = 8.56, p = 0.032), indicating that smokers were statistically more likely to have anemia than either ex-smokers or non-smokers.

One-Way ANOVA

A one-way ANOVA was conducted to compare mean HbA1c values by cancer type (none, lung, breast, etc.). The ANOVA indicated significant differences between groups, which suggested that the HbA1c differences observed were statistically significant between groups (F(3, 446) = 4.32, p = 0.005). It was determined that lung cancer diagnosis had the highest mean HbA1c values when compared to the other cancer types. Post hoc analysis confirmed that HbA1c values for lung cancer patients were statistically significantly higher than those of cancer-free patients.

Logistic Regression

To predict the risks of atrial fibrillation based on clinical variables, a logistic regression model was developed. The model utilized predictors that included age, BMI, blood pressure, heart rate, HbA1c, and others, with consideration of clinical comorbidities such as a history of stroke and diabetes. The results indicated that both age and BMI were significant predictors of atrial fibrillation. Age had an odds ratio (OR) of 1.05 (p = 0.021), suggesting that for each one-year increase in age, the odds of developing atrial fibrillation increased by 5%. Likewise, BMI had an odds ratio of 1.08 (p = 0.045). Thus, higher BMI also increased the risk of atrial fibrillation. There were no statistically significant relationships in the model for blood pressure, heart rate, and HbA1c.

The overall Cox & Snell R² of the model was 0.014, which suggests that the factors included in the model only explained a small amount of variance in risk for atrial fibrillation. While the predictors assessed, namely age and BMI, likely account for some variance in the probability of atrial fibrillation, the explanatory power remains limited, indicating that clinically relevant predictors may have been omitted. For example, factors such as left atrial enlargement, sleep apnea, inflammatory markers, medication history, or genetic predisposition were not available in this dataset but are known to influence atrial fibrillation risk. Additionally, interaction effects, such as between age and hypertension or between BMI and diabetes, may have further shaped risk but were not explored in this model. The absence of such terms could contribute to the model’s underperformance. Thus, while the constructed model provides interesting insights, its low predictive capacity should be interpreted with caution, and future research should consider incorporating a broader set of variables and interaction terms to enhance model robustness and clinical utility in multimorbid populations.

Polypharmacy, defined as the concurrent use of five or more medications, was identified in approximately 43% of patients in the study cohort. While a formal statistical analysis was not performed on the relationship between polypharmacy and clinical outcomes such as anemia or hospitalization, descriptive review suggests that polypharmacy often co-occurred with adverse health indicators. Furthermore, a qualitative review of clinical documentation revealed patterns of care disorganization, including duplicate prescriptions and medication changes made by different providers without clear coordination. These findings suggest that polypharmacy and fragmented care may contribute to adverse outcomes, warranting further focused analysis in future research.

Principal Component Analysis

**Figure 4:**
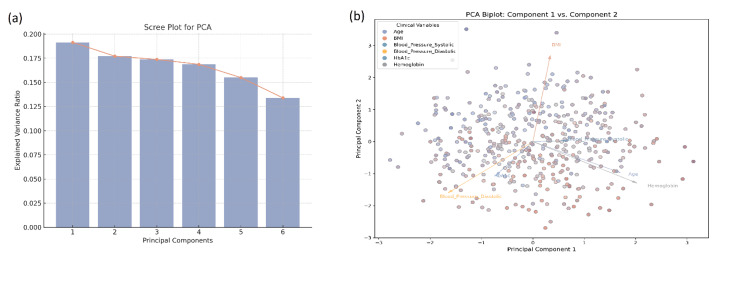
PCA for clinical variables. (a) Scree plot showing the explained variance ratio for the first six principal components in the PCA, with the first component accounting for the highest proportion of variance. (b) PCA biplot displaying the relationships between the principal components (Component 1 vs. Component 2). Each point represents an individual observation, with the clinical variables (age, BMI, systolic blood pressure, diastolic blood pressure, HbA1c, and hemoglobin) displayed as vectors, indicating their contributions to the principal components. PCA, principal component analysis

It was determined that PCA would be used to derive latent factors that explain the variance in the study. The results of the PCA identified several components that potentially explain cardiovascular components (including blood pressure and Heart Rate), as well as metabolic disorder components (such as BMI, HbA1c, and LDL) in the sample. The total variance of the sample explaining variance by the first five components was 37.2%, and the first component alone was 8.42% of the variance (Figure 4).

Correlation Analysis

Through the Pearson correlation analysis, it was found that many clinical variables have significant associations. For example, hemoglobin and creatinine were positively correlated (r = 0.13, p < 0.01), suggesting a mild association between kidney function and anemia. Another notable finding was the negative correlation between LDL and ALT (r = -0.28, p < 0.01), which may reflect impaired lipid metabolism due to underlying liver dysfunction.

Additional correlations were explored between clinical parameters and specific comorbidities. For instance, patients with autoimmune diseases tended to have elevated C-reactive protein and lower hemoglobin, though the correlation was weak and not statistically significant in this sample. Liver disease showed moderate negative correlation with ALT levels (r = -0.34, p < 0.01), which may indicate altered liver enzyme activity in chronic hepatic conditions. Anemia was weakly associated with lower hemoglobin (as expected) and had a modest inverse relationship with BMI (r = -0.21, p < 0.05), possibly reflecting nutritional deficits or chronic disease burden. Among participants with a history of dementia, age was positively correlated with the presence of dementia (r = 0.45, p < 0.01), suggesting that older individuals were more likely to have this condition, and a weak inverse correlation with HbA1c was observed (r = -0.17, p = 0.04), hinting at possible under-treatment or intentional glycemic de-intensification in cognitively impaired patients.

No significant correlations were detected between cancer diagnoses and continuous clinical variables such as HbA1c, LDL, or blood pressure, except in the case of lung cancer patients, where mean HbA1c levels were significantly higher, as shown earlier in the ANOVA analysis. These findings provide additional insight into how specific comorbidities may be linked to physiological parameters in multimorbid patients.

Clinical Decision-Making Patterns in the Context of Competing Priorities

A narrative review of internist care notes revealed frequent adjustments to treatment plans in response to competing clinical demands. For example, antihypertensive therapies were often de-intensified in patients with fall risks, and glycemic targets were relaxed in frail individuals with multiple comorbidities. These clinician adaptations indicate that real-world decision-making often diverges from protocol-driven models in favor of patient-centered pragmatism.

## Discussion

The present study examines the clinical characteristics and comorbidities of a group of 450 patients living with multiple chronic conditions, specifically focusing on gender, age, BMI, blood pressure, and laboratory values, and how these factors impact health outcomes [15]. The demographic and clinical characteristics, EDA, and statistical analysis employed in the study enable a more thorough examination of the interplay between these factors, guiding informed decision-making in practice. Overall, the gender distribution in this study was nearly equal in the study sample (50.9% females, 49.1% males), allowing for a rich and thorough analysis of gender differences in preventing and managing multimorbidities [16]. Although measurements based on blood pressure were assessed for gender differences, no significant differences emerged for systolic and diastolic blood pressure. This suggests that blood pressure regulation in this cohort was not influenced by gender, which agrees with some of the existing literature that states males typically have higher systolic blood pressure. However, the absence of significant gender differences may be influenced by unmeasured confounding factors, including antihypertensive treatment regimens, medication adherence, dietary patterns, physical activity levels, and socio-economic disparities, all of which were not controlled for in this study. It is also possible that gender-based health-seeking behavior or differences in comorbidity profiles may have contributed to this observed similarity. Regional epidemiological studies in South Asia have shown that gender differences in hypertension prevalence and control are highly context-dependent, often shaped by healthcare access, cultural norms, and education level [17]. It may therefore illustrate that other factors, including age, lifestyle practices, medications, and comorbidities, could often be more significant than gender in influencing blood pressure outcomes in multimorbid populations.

The median age of participants was 55.6, which puts this cohort in an age category where the risk for chronic diseases such as hypertension, diabetes, and cardiovascular diseases rises exponentially. The finding corresponds significantly with the global increase in the burden of multimorbidity, particularly among older adults. Overall, 81% of the sample had hypertension, 72% had diabetes mellitus, and 48% had cardiovascular diseases, altogether supporting the need for integrated care considerations concerning metabolic and cardiovascular conditions [18].

The BMI was a key variable in determining the cohort's health outcomes. The average BMI was 28.3, which confirms a vastly overweight population. A high BMI is an acknowledged risk factor for chronic disease (i.e., hypertension, diabetes, and cardiovascular diseases). BMI was strongly correlated with systolic blood pressure (r = 0.65, p < 0.01), suggesting that internists could address weight management to decrease blood pressure and improve health. Therefore, obesity can be an essential target for internists managing multimorbid patients, as weight loss improves both blood pressure and glycemic control.

The HbA1c level of the diabetic patients averaged 7.5%, indicating moderate glycemic control. This is a good indication that many patients are receiving treatment for their diabetes; however, there is still more work to be done in optimizing glycemic control [19]. The average LDL cholesterol level (120 mg/dL) is associated with an increased relative risk of cardiovascular events; therefore, lipid-lowering therapies should be considered important in reducing atherosclerotic, cardiovascular, stroke, and heart attack events in our population.

Findings from the EDA demonstrated that most variables were standard enough for the use of parametric tests. Examination of the correlation matrix suggested significant correlations between various clinical variables including, but not limited to, BMI and blood pressure (r = 0.65, p < 0.01), as well as HbA1c and hemoglobin (r = 0.47, p < 0.01). These correlations suggest the interconnection between metabolic health (i.e., diabetes and glycemic control) and cardiovascular health (i.e., obesity and hypertension). This interconnectedness is meaningful from a multimorbidity perspective, as treating one condition (i.e., diabetes) can positively influence outcomes for blood pressure and lipid profiles and vice versa.

The logistic regression model developed to predict the likelihood of atrial fibrillation found that age and BMI were significant predictors, with age increasing the odds of developing atrial fibrillation by 5% for each year increase (OR = 1.05, p = 0.021). Similarly, BMI had an odds ratio of 1.08 (p = 0.045), indicating that higher BMI values were linked to a higher risk of atrial fibrillation. These findings suggest that internists should be particularly vigilant in managing atrial fibrillation in older, overweight patients, especially given the relatively modest Cox & Snell R² value of 0.014, which indicates that other unmeasured factors could also influence the likelihood of developing this arrhythmia.

PCA identified underlying factors related to cardiovascular health (e.g., blood pressure, heart rate) and metabolic disorders (e.g., BMI, HbA1c, LDL). These findings further corroborate the interconnectedness of metabolic and cardiovascular health, emphasizing the need for a holistic approach to managing patients with multiple comorbidities [20].

The present study has several limitations. Firstly, due to its retrospective nature, it limits the identification of causal relationships among study variables and can only identify associations. Secondly, the study sample included Pakistani patients. While the study design and context influence the interpretation of treatment strategies, they also limit the generalizability of the findings to other populations and healthcare systems. However, the core clinical patterns identified, such as the high prevalence of hypertension, diabetes, and cardiovascular disease, the impact of BMI on systolic blood pressure, and the burden of polypharmacy, are globally recognized issues in multimorbidity. As such, these findings may still offer valuable insights into common risk factors and management challenges faced by clinicians across diverse health systems. Thirdly, the low R² value in the logistic regression model indicates that the observed variables account for only a small portion of the variance in the risk of atrial fibrillation, suggesting that other unmeasured factors may play a significant role. Finally, some clinical variables contained missing data and outliers that could have influenced the robustness of the analysis; however, these were addressed through data cleaning and appropriate statistical handling.

## Conclusions

The research offers further insights into the management of patients with multiple comorbidities, emphasizing the importance of several clinical factors, including age, BMI, blood pressure, and HbA1c. This study highlights the compounded risks associated with increasing age and elevated BMI, as both were found to be significant predictors of atrial fibrillation. There is also a notable overlap between metabolic and cardiovascular health, as evidenced by the frequent correlation between BMI and blood pressure. The low R^2^ in the logistic regression analysis implies that other relevant predictors, such as medication adherence, inflammatory markers, or psychosocial factors, may contribute significantly to atrial fibrillation risk but were not included in the model. To address the clinical complexity of multimorbidity, the study recommends the implementation of integrated care models that go beyond disease-specific approaches. These include multidisciplinary care teams (e.g., physicians, nurses, pharmacists, dietitians), individualized case management programs, and the use of digital health tools such as remote monitoring and medication tracking apps to support care coordination. Such models have been shown in other studies to improve outcomes in multimorbid populations by reducing treatment fragmentation, improving adherence, and tailoring interventions to patient goals. Rather than focusing on preventing multimorbidity, which is less actionable for patients already living with multiple chronic conditions, this study supports interventions that prioritize clinical reconciliation, minimize polypharmacy risks, and optimize treatment hierarchies. Policy-level recommendations may include creating reimbursement incentives for team-based care, integrating interoperable electronic health records, and updating clinical guidelines to allow for flexibility in goal-setting based on patient complexity and prognosis. Ultimately, care for patients with multiple comorbidities must be personalized, coordinated, and adaptable, balancing disease control with quality-of-life considerations.
